# Engrailed 1 overexpression as a potential prognostic marker in Lower Grade Glioma

**DOI:** 10.7717/peerj.7414

**Published:** 2019-09-16

**Authors:** Jin Zhu, Yu-Qi Zhang

**Affiliations:** 1 Beijing Institute of Functional Neurosurgery, Department of Functional Neurosurgery, Xuanwu Hospital, Capital Medical University, Beijing, China; 2 Department of Neurosurgery, Yuquan Hospital, Tsinghua University, Beijing, China

**Keywords:** Engrailed 1, Lower grade glioma, Biomarker, Oncogene

## Abstract

**Background:**

Engrailed 1 (EN1), as a member of homeobox-containing transcription factors, participates in the development of the brain. High expressions of EN1 exist in various tumors. However, the role of EN1 in lower grade glioma (LGG) is still unknown.

**Methods:**

Coefficients of Cox regression were examined by data mining among 13 cancer types using OncoLnc to validate EN1 expressions in LGG patients from The Cancer Genome Atlas database (TCGA). Bioinformatic analysis was performed by using R2 and the UCSC Xena browser based on the data from 273 glioma cases in GSE16011 from GEO datasets and 530 cases of LGG patients in TCGA. Cases in GSE16011 were divided into two groups according to IDH1 mutation status. Cases in TCGA-LGG were classified to subtypes according to histopathological results, IDH1 mutation status and 1p19q status. The Kaplan–Meier survival curves were performed to analyze the relationship between EN1 expressions and clinicopathological characteristics and survival time respectively.

**Results:**

Cox regression results showed that LGG was ranked statistically first among 13 different cancer types according to the false discovery rate (FDR) correction. Results from GSE16011 showed that: glioma, LGG and LGG with IDH1 mutation patients with high EN1 expressions had significantly shorter 5, 10, and 15-year overall survival time (OS) (*p* < 0.001). Similar results from TCGA-LGG showed that LGG patients with high EN1 expressions had significantly shorter 15-year OS, irrespective of IDH1 mutation and 1p19q co-deletion (*p* < 0.001). The astrocytoma subgroup showed highest levels of EN1 expression and shortest 5, 10 and 15-year OS compared with oligoastrocytoma and oligodendroglioma (*p* < 0.05).

**Conclusion:**

EN1 can be used as a prognostic marker in LGG patients, combined with IDH1 mutation and 1p19q co-deletion.

## Introduction

Gliomas are the most common primary brain tumors and can be divided into four grades based on the classification scheme of the World Health Organization (WHO). The lower grade gliomas (LGGs) constitute 20% of all gliomas, with various biological features and comparatively good prognosis (Ruiz & Lesser, 2009). LGGs traditionally include WHO grade I and grade II gliomas, whose main pathological types include astrocytoma, oligoastrocytoma, oligodendroglioma and so on (Ceccarelli et al., 2016). The clinical symptoms usually present with seizures and other neurological disorders, depending on the size and location of the tumor. Although with benign pathology, LGGs may sometimes even transform to high grade gliomas (HGGs). With combined and available treatments, 10-year survival rate of patients with LGGs is still lesser than 50% (Shaw, Scheithauer & O’Fallon, 1997).

With the development of research on the causes and mechanism of glioma, lots of oncogenes and tumor-suppressor genes have been found. They can promote or inhibit the growth and progress of the tumor through various pathways. The genetically-targeted treatment becomes a novel method nowadays (Karsy et al., 2017). IDH1 mutation and loss of 1p/19q in LGG patients usually comply to longer overall survival (OS) (Izquierdo et al., 2018).

Engrailed 1 (EN1), a neural-specific transcription factor, plays a crucial role in the development of many tissues and organs (Izquierdo et al., 2018). EN1 expression persists not only in the dopaminergic neurons of the substantia nigra but also the ventral tegmental area not just in the embryonic stage but also during the whole individual’s life (Alvarez-Fischer et al., 2011). High EN1 expression has been found in patients with breast tumors (Beltran, Graves & Blancafort, 2014), salivary gland adenoid cystic carcinoma (Bell et al., 2012) and adenoid cystic carcinoma (Drier et al., 2016), with increased recurrence and mortality rate. However, the relationship between EN1 and LGGs has not been reported.

In this study, by data mining in large micro-array datasets, we characterized the expression profile of EN1 in LGGs with histological subtypes, IDH1 mutation and 1p19q co-deletion status to assess the associations between EN1 expression and OS.

## Material and Methods

### Datasets

Glioma patients were assessed with the data in GSE16011 from the GEO dataset. Of all the 284 cases in GSE16011, 273 were glioma and 117 were LGG. 46 cases were LGG with IDH1 mutation, and 45 cases were LGG without IDH1 mutation.

The LGG cohort in TCGA database (TCGA-LGG) was obtained from the UCSC Xena browser (https://xenabrowser.net), which included 530 cases of LGG with genomic and clinical data. The genomic dataset contained IDH1 status, chromosome 1p19q deletion status, EN1 mRNA expression and so on. The clinical dataset contained demographic, survival rate, histological and pathological information.

### Bioinformatic analysis of the association between EN1 expression and OS in patients with glioma and LGG

**Figure 1:**
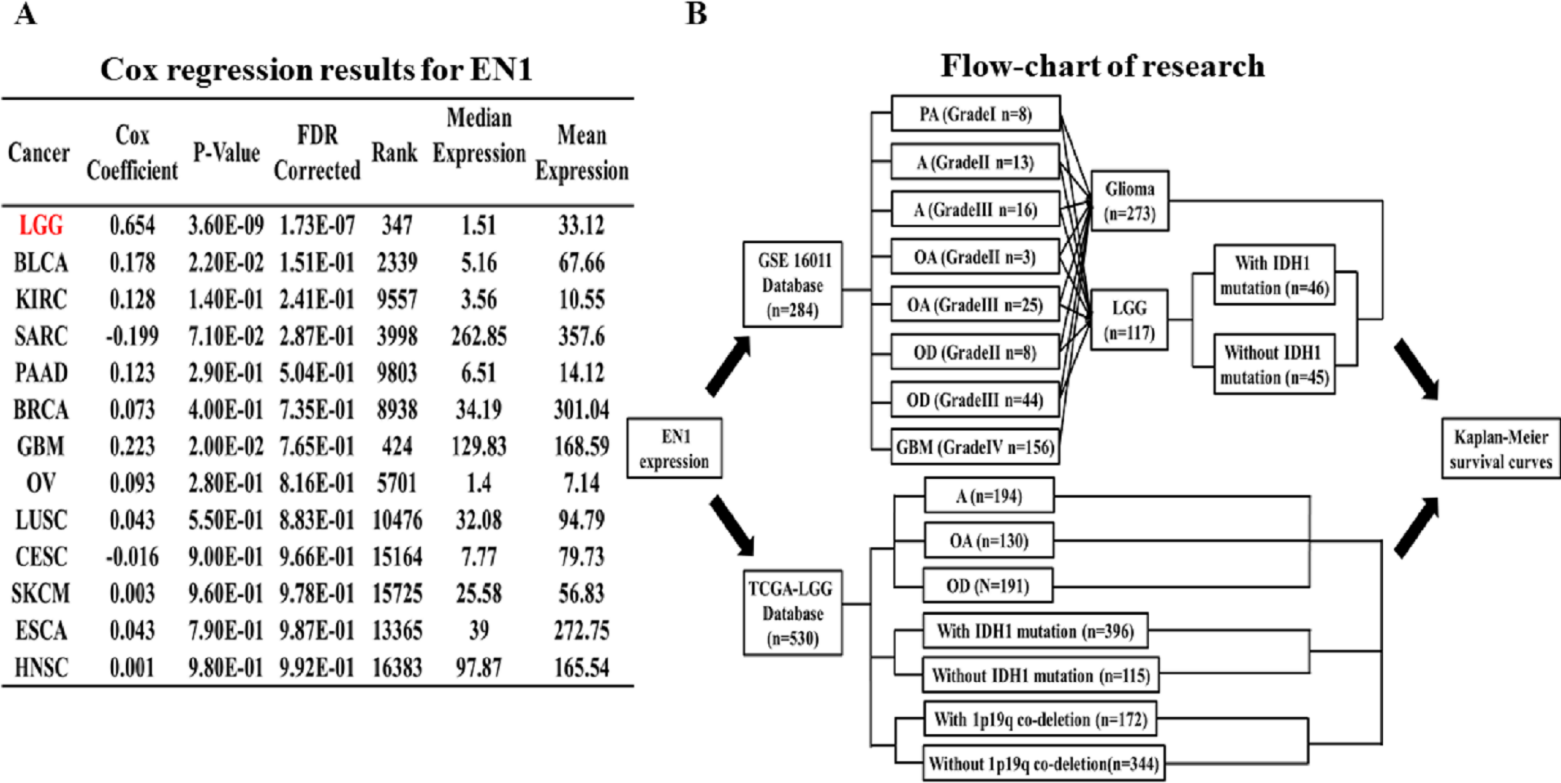
Coefficients of Cox regression of EN1 in different cancers and flow-chart of research. (A) Coefficients of Cox regression were examined by data mining among 13 TCGA cancer types using OncoLnc (http://www.oncolnc.org). (B) A flow-chart showed detailed information about cases in two datasets and methods of research.

Coefficients of Cox regression were examined by data mining among 13 TCGA cancer types using OncoLnc (http://112www.oncolnc.org) to compare EN1 expression in different tumors (Fig. 1A).

The association between EN1 expression and OS (5, 10 and 15-years) in glioma and LGG patients was assessed with data in GSE16011. The R2 web-based application (http://r2.amc.nl) was used to generate Kaplan–Meier survival curves of data in GSE16011. Of the 273 qualified glioma cases in GSE16011, 156 were glioblastoma multiforme (GBM), 117 were LGG. Survival data of LGG with or without IDH1 mutation was extracted for analysis. Kaplan–Meier curves of OS were generated by using the auto-select best cutoff (Fig. 1B).

Survival data of the LGG subgroup in the TCGA dataset was analyzed through the UCSC Xena browser. Three grouping methods were selected: histological type, IDH1 mutation and 1p19q co-deletion. Gene expression data was extracted to compare the differences in EN1 expression among different subtypes. Kaplan–Meier survival curves were generated to analyze EN1 expression and OS (Fig. 1B).

### Statistical analysis

Coefficients of Cox regression were examined using OncoLnc *P* values of all the cancer types and they were corrected by false-discovery rate (FDR) and FDR <0.25 was considered statistically significant. Kaplan–Meier survival analysis was performed by using R2 web-based platform and the UCSC Xena browser. *P* < 0.05 is considered statistically significant.

## Results

### EN1 expression in LGG is most distinct among all known tumor types

Cox regression analysis was performed in the TCGA datasets. The results showed that EN1 expression in LGG was ranked statistically first among 13 different cancer types according to FDR correction (Fig. 1A).

### High EN1 expression might be an indicator of poor OS in patients with glioma and LGG

**Figure 2:**
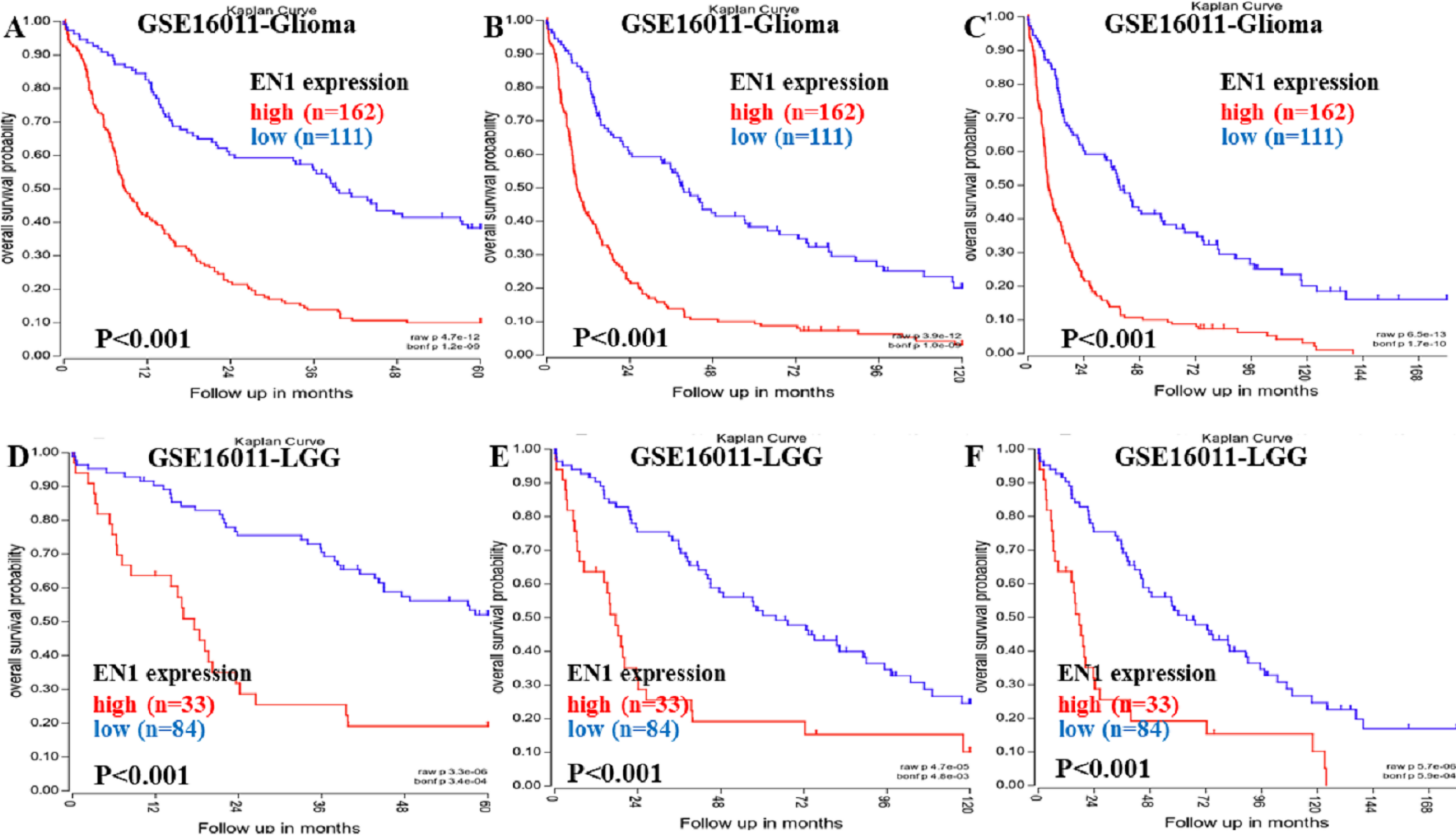
Kaplan–Meier curves of OS in glioma and LGG patients with high or low EN1 expression in GSE16011. (A–C) Kaplan-Meier curves of 5-year OS (A), 10-year OS (B) and 15-year OS (C) in the glioma patients with high or low EN1 expression in GSE16011. (D–F) Kaplan-Meier curves of 5-year OS (D), 10-year OS (E) and 15-year OS (F) in the LGG patients with high or low EN1 expression in GSE16011. OS curves were generated by using auto-select best cutoff. High or low EN1 expression are showed in red or blue color respectively. Analysis was performed using R2.

Kaplan–Meier survival analysis was chosen to explore the association between EN1 expression and 5, 10 and 15-year OS in patients with glioma through data mining in R2 using data in GSE16011 (Figs. 2A–2F).

Higher expression of EN1 in glioma correlated with shorter patient 5, 10 and 15-year OS according to R2 (*p* < 0.001) (Figs. 2A–2C). LGG patients with higher expression of EN1 also had shorter 5, 10 and 15-year OS (*p* < 0.001) (Figs. 2D–2F).

### High EN1 expression might be an indicator of poor OS in LGG patients with IDH1 mutation

**Figure 3:**
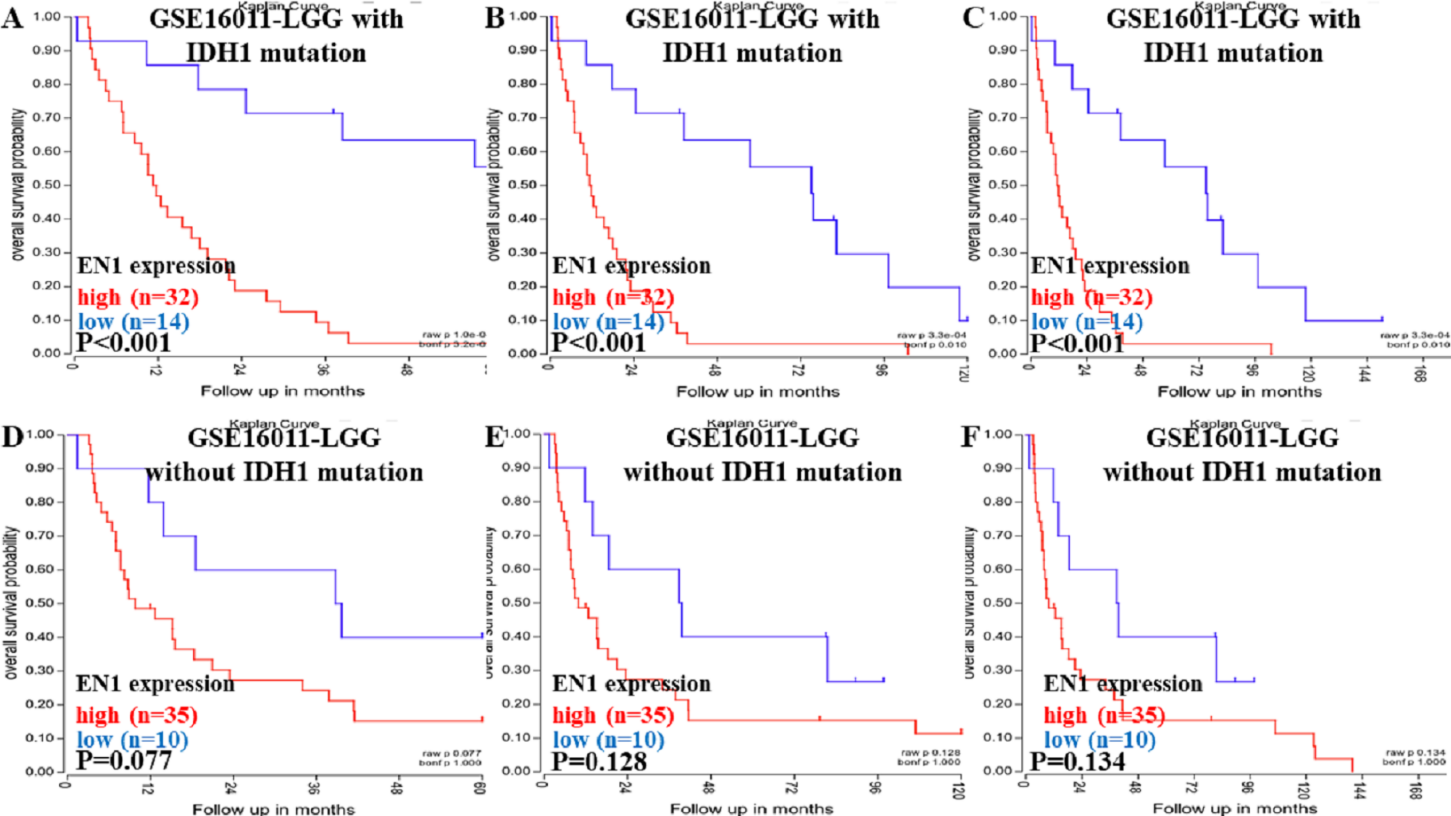
Kaplan-Meier curves of OS in LGG patients with or without IDH1 mutation in GSE16011. (A–C) Kaplan-Meier curves of 5-year OS (A), 10-year OS (B) and 15-year OS (C) in the LGG patients with IDH1 mutation in GSE16011. (D–F) Kaplan-Meier curves of 5-year OS (D), 10-year OS (E) and 15-year OS (F) in the LGG patients without IDH1 mutation in GSE16011. OS curves were generated by using auto-select best cutoff. High or low EN1 expression are showed in red or blue color respectively. Analysis was performed using R2.

Kaplan–Meier survival analysis was chosen to explore the association between EN1 expression and 5, 10 and 15 year OS in LGG patients with/without IDH1 mutation through data mining in R2 using data in GSE16011 (Figs. 3A–3F).

Higher expression of EN1 in LGG with IDH1 mutation correlated with shorter patient 5, 10 and 15- year OS (*p* < 0.001) (Figs. 3A–3C). It seemed that higher expression of EN1 in LGG with IDH1 mutation correlated with shorter patient 5, 10 and 15- year OS, but there was no statistical significance (*p* > 0.05) (Figs. 3D–3F).

### EN1 expression in different histological subtypes of LGG

**Figure 4:**
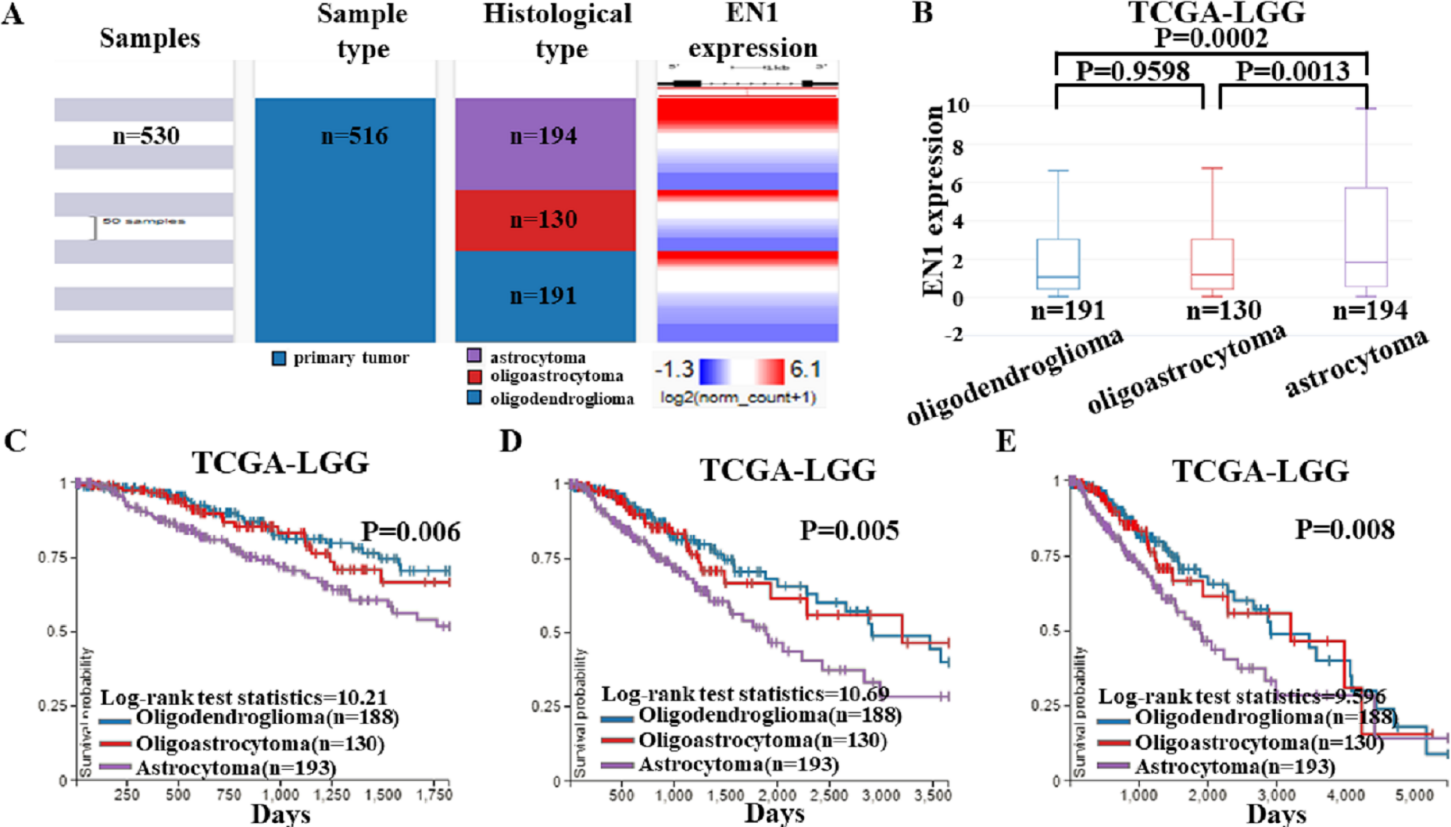
EN1 expression and Kaplan-Meier curves of OS in different histological subtypes in TCGA-LGG. (A–B). Heat map and the corresponding box plots of EN1 expression profile in various histological subtypes of LGG based on TCGA-LGG. (C–E). Kaplan-Meier curves of 5-year OS (C), 10-year OS (D) and 15-year OS (E) in the subtypes of LGG with high or low EN1 expression in TCGA-LGG. OS curves were generated by setting median EN1 expression as cutoff. Analysis was performed using the UCSC Xena browser.

To detect EN1 expression in different histological subtypes of LGG and association between them, we examined the expression profile of EN1 in various histological subtypes of LGG based on TCGA-LGG (Fig. 4A).

The box plots showed highest EN1 expression in patients with astrocytoma (*p* < 0.001), but no statistical differences had been found between oligoastrocytoma and oligodendroglioma patients (*p* > 0.05) (Fig. 4B).

Kaplan–Meier survival analysis was also generated to detect the association between 5, 10, and 15- year OS in patients with three histological subtypes. Along with EN1 expression, patients with astrocytoma had the shortest OS (*p* < 0.05). Remarkably, oligoastrocytoma and oligodendroglioma patients had similar longer OS. These results showed that EN1 may be a prognostic maker for different histological subtypes, especially for astrocytoma (Figs. 4C–4E).

### EN1 expression in LGG patients with/without IDH1 mutation from TCGA-LGG dataset

**Figure 5:**
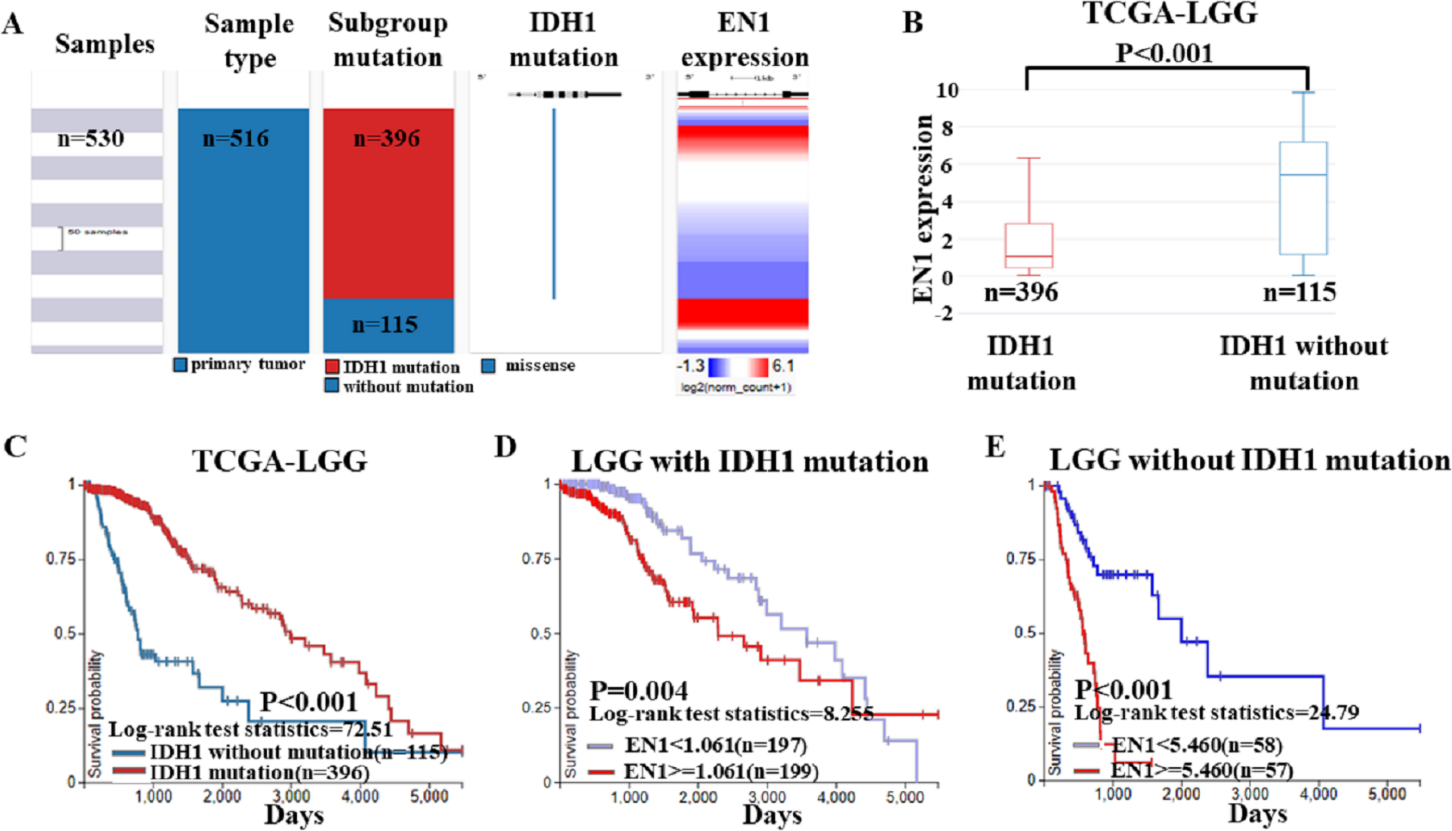
EN1 expression in LGG patients with or without IDH1 mutation in TCGA-LGG. (A) The heat map of EN1 expression in genomic subclassifications of LGG, including patient samples, IDH1 mutation status and EN1 mRNA expression level. (B) The corresponding box plots of EN1 expression profile in LGG patients with or without IDH1 mutation based on TCGA-LGG. (C) Kaplan-Meier curves of 15-year OS in the subtypes of LGG with or without IDH1 mutation. (D) Kaplan-Meier curves of 15-year OS in patients with high or low EN1 expression in IDH1 mutation group. (E) Kaplan-Meier curves of 15-year OS in patients with high or low EN1 expression in no IDH1 mutation group. OS curves were generated by setting median EN1 expression as cutoff. High or low EN1 expression are showed in red or blue color, respectively. Analysis was performed using the UCSC Xena browser.

To further confirm the findings of data from the GEO dataset, we classified the cases in TCGA-LGG dataset into two groups: LGG with IDH1 mutation and LGG without IDH1 mutation. Heat map and the corresponding box plots showed that LGG with IDH1 mutation patients had a lower EN1 expression (Figs. 5A–5B).

Kaplan–Meier curves was generated and showed that the LGG with IDH1 mutation had significantly more 15-year OS (*p* < 0.001) (Figs. 5B–5C).

The negative correlation between EN1 expression and OS existed in both LGG irrespective of IDH1 mutation: Lower expression of EN1 correlated with improved patient 15-year OS (*p* < 0.05) (Figs. 5D–5E).

### EN1 expression in LGG patients irrespective of 1p19q co-deletion from TCGA-LGG dataset

**Figure 6:**
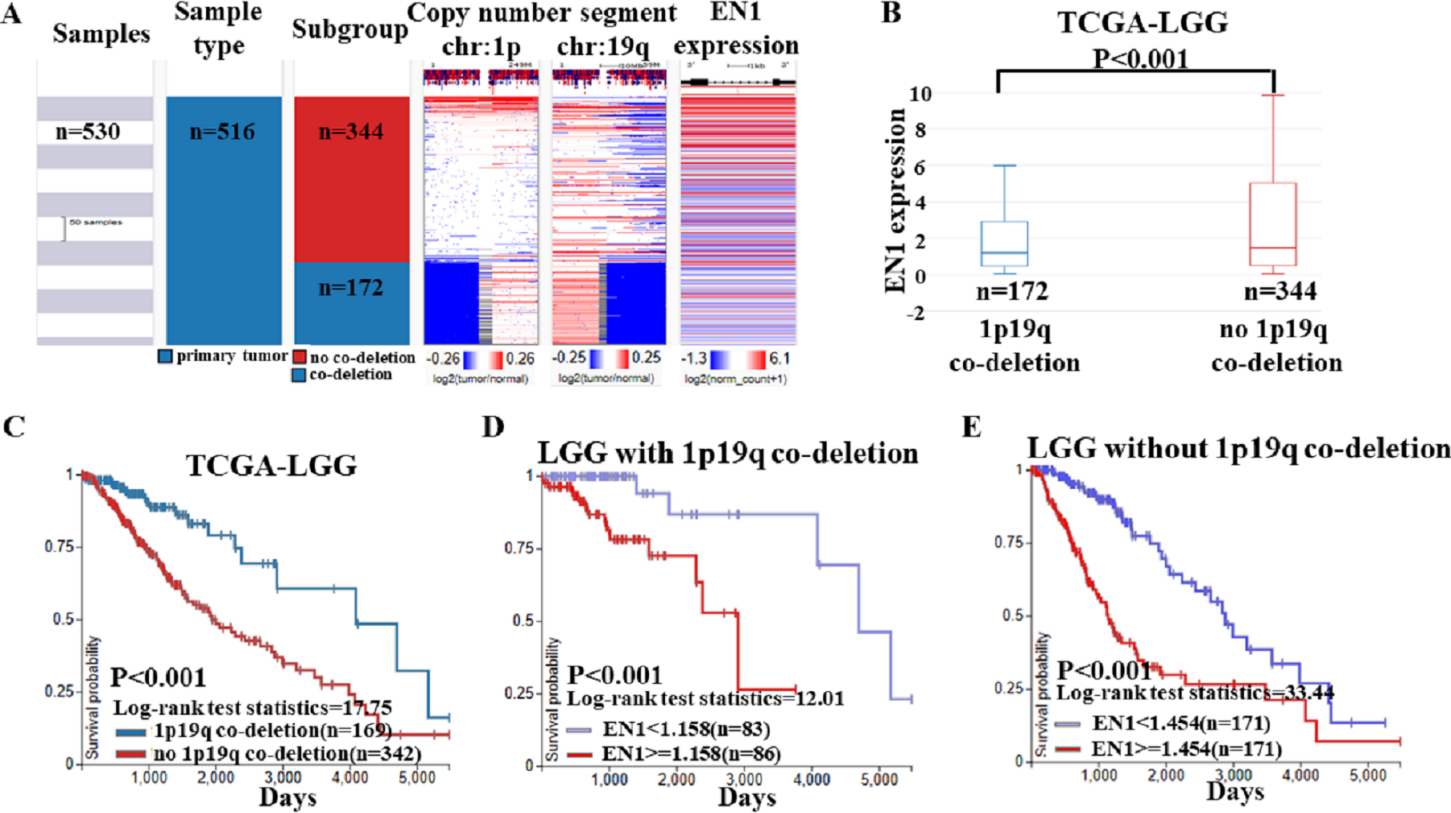
EN1 expression in LGG patients with or without 1p19q co-deletion in TCGA-LGG. (A) The heat map of EN1 expression in genomic subclassifications of LGG, including patient samples, 1p19q DNA copy number status and EN1 mRNA expression level. (B) The corresponding box plots of EN1 expression profile in LGG patients with or without 1p19q co-deletion based on TCGA-LGG. (C) Kaplan-Meier curves of 15-year OS in the subtypes of LGG with or without 1p19q co-deletion. (D) Kaplan-Meier curves of 15-year OS in patients with high or low EN1 expression in 1p19q co-deletion group. (E) Kaplan-Meier curves of 15-year OS in patients with high or low EN1 expression in no 1p19q co-deletion group. OS curves were generated by setting median EN1 expression as cutoff. High or low EN1 expression are showed in red or blue color, respectively. Analysis was performed using the UCSC Xena browser.

Based on the data of 1p19q status and EN1 expression, a heat map was generated. Heat map and the corresponding box plots showed that: LGG with 1p19q co-deletion expressed lower levels of EN1 in comparison with the no 1p19q co-deletion group (*p* < 0.001) (Figs. 6A–6B).

The results of Kaplan–Meier survival analysis showed that: (1) 15- year OS in LGG patients with 1p19q co-deletion is significantly higher than the ones without 1p19q co-deletion (*p* < 0.001) (Fig. 6C); (2) Higher EN1 expression is consistent with shorter 15- year OS in LGG patients with/without 1p19q co-deletion (*p* < 0.001) (Figs. 6D–6E).

## Disscussion

EN1, a murine homologue of the Drosophila homeobox gene engrailed (EN), is required for midbrain and cerebellum development and dorsal/ventral patterning of the limbs. EN1 may form a complex or directly play its role through development (Bilovocky et al., 2003). Since first reported in 1926, Drosophila, flies and other animal experiments were selected to explore its function (Higashijima et al., 2004; Frankel et al., 2010; Barolo, 2012). The results showed that EN1 mutation may reduce adrenergic and serotonergic neurons of the vertebrate brainstem (Kumar et al., 2009).

Expression of EN1 can be found in lots of structures including the central nervous system (Plummer, De Marchena & Jensen, 2016). In schizophrenic patients, EN1 was discovered to be associated with antipsychotic response (Webb et al., 2008). In patients with tumors, high EN1 expression was associated with reduced survival time (Webb et al., 2008). In mice with loss of EN1, Parkinson disease-like motor or non-motor symptoms will appear, which implies that EN1 probably can be a therapeutic target for Parkinson disease (Rekaik et al., 2015). EN1 may also play a critical role in the modulation of calvarial osteoblast differentiation and proliferation to ensure proper skull vault formation (Zheng et al., 2015)

EN1 may promote the proliferation, migration and multinucleation of cancer cells via transcriptional activation of HDAC8, UTP11L and ZIC3 (Kim et al., 2018). The interference peptides (EN1-iPeps) that selectively inhibit EN1 activity can be used for the treatment of aggressive basal-like triple negative breast carcinomas (Gandhi, Blancafort & Mancera, 2018). EN1-iPeps inhibit interactions between EN1 and its binding partners such as glutamyl-prolyl tRNA synthetase (EPRS) (Beltran, Graves & Blancafort, 2014). Combined with traditional anti-tumor drugs, it has an obvious inhibitory effect.

The EN1 expression in human glioma and subtypes has not been reported. We chose two online databases to analyze EN1 expressions in glioma and LGG. LGG were grouped according to histological types, IDH1 mutation status and 1p19q co-deletion status. R2 results showed that lower EN1 expression was significantly correlated with longer 5, 10 and 15-year OS in glioma and LGG patients in the GEO dataset. Kaplan–Meier survival curves were similar in LGG patients with IDH1 mutation and without mutation; although no significant difference was found in the latter group.

The TCGA-LGG data analysis results were consistent with R2 results. EN1 expression in LGG was ranked statistically first among all 13 different cancer types according to the FDR correction. The astrocytoma had the highest EN1 expression and shortest OS compared to oligoastrocytoma and oligodendroglioma. Compared with the corresponding group, LGG with IDH1 mutation or 1p19q co-deletion had a lower EN1 expression and a longer OS. Whether with IDH1 mutation or not, LGG patients with lower EN1 expression had significantly more 15-year OS. The similar negative correlation existed in patients with/without 1p19q co-deletion.

Therapy for LGG is a challenge for neurosurgeons. Previous studies showed that IDH1 mutation and 1p19q co-deletion may be related to the therapeutic effect in LGG patients and has been used as prognostic indicator (Mondesir et al., 2016). By data analysis in GSE16011 and TCGA-LGG, the consistent results showed that EN1 might be an indicator of favorable OS in glioma, especially in LGG. Combined with IDH1 and 1p19q, EN1 may be used for preoperative and postoperative evaluation. If EN1 is selected as a novel gene treatment target for LGG patients, OS might be prolonged in the future.

Our findings offer some evidence for EN1 effect on survival rate in LGG patients with different status. Because of limited technological methods, we cannot explore the specific carcinogenic pathway of EN1. Further studies would be necessary to elucidate the underlying mechanism of EN1.

## Conclusions

In conclusion, as the first research of EN1 in LGG is shown, these results support the importance and specificity of EN1 effect on survival rate in LGG patients with/without IDH1 mutation and 1p19q co-deletion. EN1 can be a potential indicator of favorable OS.

## Additional Information and Declarations

### Data Availability

The following information was supplied regarding data availability: The R2 web-based application (http://r2.amc.nl) was used to generate Kaplan–Meier survival curves of data in GSE16011. Cox coefficients were examined by data mining among 13 TCGA cancer types using OncoLnc (http://www.oncolnc.org/) to compare EN1 expression in different tumors. LGG cohort in TCGA database (TCGA-LGG) was obtained from the UCSC Xena browser: https://portal.gdc.cancer.gov/projects/TCGA-LGG.
